# A Curriculum Innovation on Writing Simulated Patient Cases for Communication Skills Education

**DOI:** 10.15766/mep_2374-8265.11068

**Published:** 2021-01-12

**Authors:** April R. Christensen, Carla L. Spagnoletti, Rene N. Claxton

**Affiliations:** 1 Assistant Professor, Department of Medicine, and Associate Program Director for the Hospice and Palliative Medicine Fellowship, Mayo Clinic; 2 Professor, Department of Medicine, University of Pittsburgh Medical Center; Director of the Academic Clinician-Educator Scholars (ACES) Fellowship in General Internal Medicine and Director of the Master's and Certificate Programs in Medical Education, Institute for Clinical Research Education, University of Pittsburgh; 3 Associate Professor and Program Director of the Hospice and Palliative Medicine Fellowship, Department of Medicine, University of Pittsburgh Medical Center

**Keywords:** Communication Training, Communication Skills, Simulated Patients, Curriculum Development, Case Writing, Flipped Classroom, Simulation

## Abstract

**Introduction:**

Facilitated communication practice with simulated patients (SPs) is a highly effective form of communication training. Unfortunately, little guidance exists on writing SP cases.

**Methods:**

We created a curriculum composed of a case-development workbook and case-writing session with input from national communication educators. In November 2017, we implemented the curriculum in a Teaching Communication Skills course for medical educators. Educators divided into four groups to write cases. Primary outcome was the number of criteria that cases fulfilled. Secondary outcomes were SP evaluation and educator-reported confidence and satisfaction.

**Results:**

Seventeen medical educators (including 15 fellows) completed the curriculum. Four new cases were analyzed against 24 criteria and compared to eight cases written by educators following a previous curriculum. An SP evaluated ease of portrayal for all 12 cases on a 5-point Likert scale (1 = *poor,* 5 = *excellent*). Educators completed precurriculum and postcurriculum surveys. Compared to the previous curriculum, cases based on the new curriculum incorporated 26% more case criteria (70% or 16.8 criteria/case vs. 96% or 23.0 criteria/case, *p* < .01). Ease-of-portrayal rating improved but did not differ statistically (mean: 2.8 vs. 4.5, *p* = .11). A moderate correlation was found between number of included case criteria and Likert-scale rating (*r*_s_ = .61, *p* = .03). Pre- and postcurriculum, educators reported significant increases in confidence (mean: 1.9 vs. 4.0, *p* < .01) and high curricular satisfaction (mean: 4.8).

**Discussion:**

A case-development workbook and case-writing session increased the quality of newly developed SP cases as assessed by prespecified case criteria.

## Educational Objectives

By the end of this module, participating medical educators will be able to:

1. Describe the four steps to writing a simulated patient case.
2. Write two to three learning objectives for a new case that meet all SMART criteria.
3. In coordination with three to four other medical educators, develop a new simulated patient case that fulfills at least 22 of the 24 criteria.
4. Describe the process of case review prior to implementation in an educational setting.

## Introduction

Patient communication is a crucial skill that significantly impacts how patients cope with illness.^1–5^ A highly effective form of communication training for physicians is supervised practice with simulated patients (SPs), trained individuals who portray patients based on written cases and provide verbal and emotional feedback to learners.^6–12^ While standardized patients portray patients in a uniform manner and are often used for formal testing, SPs adapt to learners’ needs during the interaction with input from the session facilitator and are used only to provide formative feedback.^12^ Training with SPs has been shown to improve physicians’ ability to communicate serious news, express empathy, and explore patients’ concerns.^7–9^

Despite the importance of communication training to patient outcomes, little guidance exists on how to write effective SP cases.^10,11,13,14^ Although a few published recommendations exist on case development for standardized patients, these focus solely on cases for objective structured clinical examinations and do not include instruction on developing cases for formative feedback in communication training.^11,13,14^ When we surveyed communication educators nationally, we found that early educators struggled to write cases given the lack of clear guidelines. Furthermore, over 90% of respondents in our national needs assessment reported interest in learning more about writing SP cases (A.R. Christensen, R. N. Claxton, unpublished data, 2017).

To address these concerns, we partnered with local and national communication educators to develop an SP case-writing curriculum. We aimed to standardize a workbook-based curriculum that could be utilized by medical educators, which we then implemented in a Teaching Communication Skills course designed for health professions postdoctoral medical educators enrolled in the master of medical education degree at the University of Pittsburgh. We postulated that the interactive workbook-based curriculum would be more effective at teaching the necessary components of an SP case than a previous lecture-based curriculum. The primary outcome was the number of prespecified case criteria that educators’ cases fulfilled. Secondary outcomes were case quality on ease of portrayal as evaluated by an experienced SP and educator-reported attitudes, confidence, and satisfaction.

## Methods

### Subjects

We implemented the curriculum with medical educators in the Teaching Communication Skills course at the University of Pittsburgh. Educators consisted primarily of fellow-level and junior faculty physicians. This course was selected because it had an existing teaching session on writing SP cases; thus, the new curriculum could be compared to the existing curriculum. The University of Pittsburgh Institutional Review Board approved the educational innovation.

### Curriculum Development

The previous 3-hour curriculum included a 30-minute lecture followed by a 90-minute breakout session in small-group format with real-time case-writing practice. During the case-writing portion, experienced SPs were available to the groups for their input. They provided feedback on whether the case information was sufficient for SP preparation and on how learners might misinterpret the case. In the final 60 minutes of the session, the written cases were acted out by SPs, and the small groups debriefed on the challenges of the writing process. The groups edited their cases based on the debrief and submitted finalized versions within a week.

The new curriculum consisted of a workbook guiding case development (Appendix A) that educators reviewed and completed prior to the in-class session, followed by a 3-hour in-class session. We developed the workbook in accordance with the six-step approach to curriculum development.^15^ The workbook was based on the available literature for writing standardized patient cases, the Association of Standardized Patient Educators standards on case development, *MedEdPORTAL*'s criteria for case submission, and examples of SP cases written by communication experts.^16–19^ The workbook reviewed four steps of writing a case: (1) educational aims, (2) clinical situation, (3) patient history, and (4) case instructions. An example case demonstrated each component, and space was provided for development of the educator's own case.

Based on the workbook and *MedEdPORTAL*'s criteria for case submission, the curriculum instructor developed a checklist of 24 case criteria that the coauthors reviewed for face validity (Appendix B). We divided the case criteria into three subgroups: educational aims, case information, and case instructions.

Once the workbook and the checklist of case criteria had been drafted, they were evaluated by five local and national communication and SP educators. The educators included two palliative care physicians, one general medicine physician, the director of a standardized patient program at the University of Pittsburgh, and the owner of a simulated patient company with almost 20 years of experience working as an SP and 5 years of experience training SPs. Their feedback helped refine the workbook.

### Implementation

We provided educators with paper versions of the precurriculum surveys that they completed in class (Appendix C). The instructor then distributed both electronic and hard copies of the finalized workbook. We asked educators to review the workbook in the next 5 days, prior to the in-class session, and write ideas for an SP case that could be further developed in class.

At the beginning of the 3-hour in-class session, the instructor answered questions on the workbook content. The educators then divided into four groups of four to five educators and spent 2 hours writing a new SP case. During the 2 hours, the instructor was available to answer questions. Afterwards, the educators and instructor spent 30 minutes debriefing as a large group and processing challenges in the case-writing process; they also discussed how the target learner had influenced the writing and what steps they had taken to ensure the case was realistic. The educators then completed a paper version of the postcurriculum survey (Appendix D). A facilitator guide is provided in Appendix E.

### Measures

After the in-class session, a course instructor provided deidentified copies of the four cases from the current year and all eight cases from the last 3 years of the previous curriculum to the curriculum instructor. The primary outcome was the total number of 24 case criteria on the developed checklist that the SP cases included; we compared cases from the current curriculum to cases from the previous curriculum. In addition, we analyzed the total number of case criteria by each of the subgroups: educational aims, case information, and case instructions.

Two blinded evaluators (the instructor and an expert SP) separately reviewed over a third of the cases to assess interrater reliability and ensure standard application of the 24 case criteria. We incorporated an SP as one of the evaluators based on her expertise in this area gained from more than 20 years of experience working as an SP and many years training SPs. During the review, the instructor and SP were both blinded as to whether a case had been written based on the previous lecture-based curriculum or the new workbook-based curriculum. With 93% concordance between reviewers, the instructor and SP came to consensus on the remaining 7% of differences in evaluation. The instructor then completed assessment of the remaining cases.

The SP separately evaluated the quality of all cases for ease of portrayal on a 5-point Likert scale (1 = *poor,* 5 = *excellent*). Independent SP review was selected as a secondary outcome to assess correlation with the developed case criteria checklist and because SP review is an essential component of the case-development process prior to implementation in the educational setting.

Other secondary outcomes included educators’ (1) pre- and postcurriculum attitudes toward case development, (2) pre- and postcurriculum confidence in writing SP cases, and (3) satisfaction with the curriculum's clarity and effectiveness. We assessed these outcomes via 5-point Likert scales (1 = *strongly disagree,* 5 = *strongly agree*) in the deidentified pre- and postcurriculum surveys.

On the precurriculum survey, we collected information on prior experience with communication teaching and with writing SP cases. Open-ended feedback on the workbook and in-class session was collected on the postcurriculum survey. We obtained demographic information from the class registrar rather than from the surveys to protect educator confidentiality.

### Statistical Analysis

We manually entered data into a REDCap-based data-collection instrument and completed analysis with Stata 15 SE (StataCorp). The primary outcome and subgroup outcomes were analyzed with the Fisher exact test. The SP-evaluated Likert ratings for the cases were assessed with the Wilcoxon rank sum test. Correlation between the number of included case criteria and SP-evaluated Likert ratings was analyzed with Spearman rank correlation. Secondary outcomes of pre- and postcurriculum attitudes and confidence were analyzed with the sign test. Descriptive statistics were used to report baseline characteristics of the educators.

## Results

All 17 educators in the class completed the new curriculum, including the pre- and postcurriculum surveys and the in-class writing session. Educator demographics are shown in Table 1. Only one educator had prior experience teaching with SPs, and none had prior experience writing SP cases. Forty-seven educators had completed the previous lecture-based curriculum between 2014 and 2016.

**Table 1.**
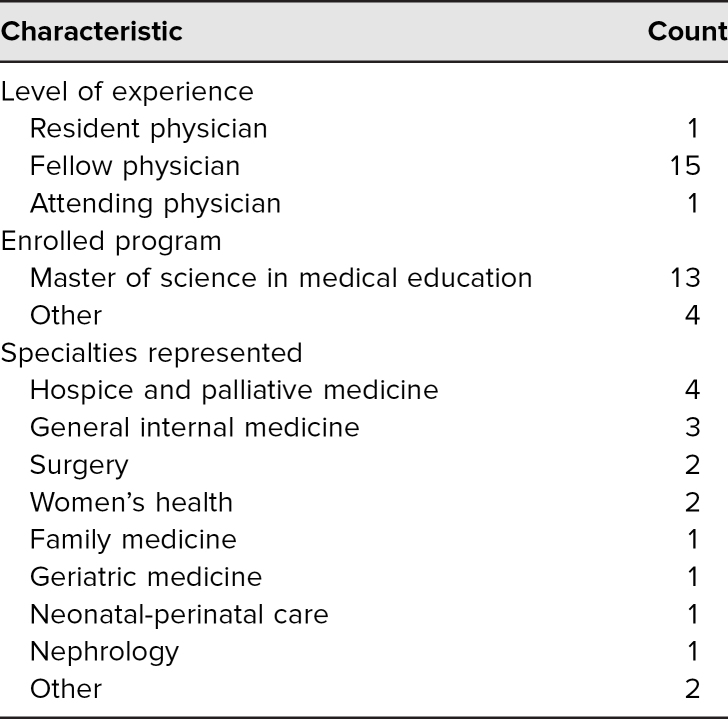
Baseline Characteristics of Medical Educators

Four cases from the new curriculum and eight cases from the last 3 years of the previous curriculum were included in the analysis. Cases developed from the new curriculum incorporated 26% more case criteria overall compared to previous years (70% or 16.8 criteria/case vs. 96% or 23.0 criteria/case, *p* < .01). Only new-curriculum cases included 21 or more of the criteria (Figure). There was a statistically significant increase with the new curriculum in case criteria included across all three subgroups: educational aims, case information, and case instruction (Table 2).

**Figure.**
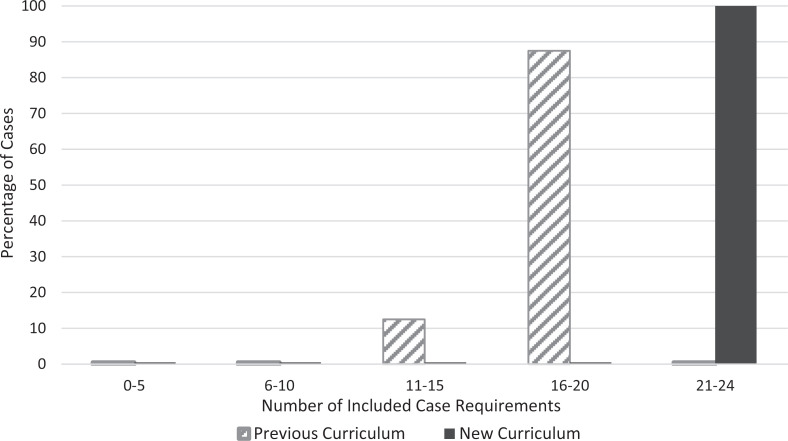
Percentage of standardized patient cases including case criteria.

**Table 2.**
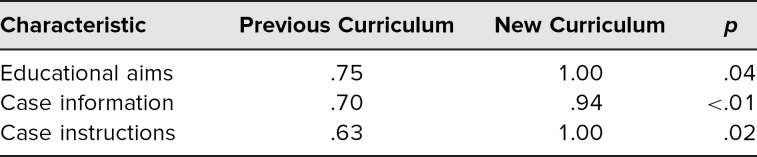
Proportion of Case Criteria Present by Subgroup

The most commonly missed criteria from the previous curriculum were those most directly applicable to communication—the patient's medical literacy level and values relevant to decision-making. These were documented in 0% and 12% of cases, respectively. With the new curriculum, medical literacy was documented in 75% of cases, and patient values were documented in 50%.

Cases based on the previous curriculum also missed several educational aims. The educational goal for the case was documented in only 50%, and the training level of the learner was documented in 75%. All of the educational aim criteria were documented in cases based on the new curriculum.

Although the new-curriculum cases were rated higher on the 5-point Likert scale for ease of portrayal as assessed by the SP, this difference was not statistically significant (mean rating: 2.8 vs. 4.5, *p* = .11). However, a moderate correlation was found between the number of included case criteria and the Likert-scale rating (*r*_s_ = .61, *p* = .03).

Educators’ confidence in writing SP cases increased after the curriculum, as rated on a 5-point Likert scale (mean: 1.9 vs. 4.0, *p* < .01). Both pre- and postcurriculum, educators agreed that writing SP cases was an important skill for clinician educators (precurriculum mean: 4.0 vs. postcurriculum mean: 4.2, *p* = .36). Overall, educators reported high satisfaction on the clarity (mean: 4.5) and effectiveness (mean: 4.8) of the curriculum.

In the postcurriculum survey, the most requested change to the in-class session was increasing the large-group debrief time to allow every group to share the case it developed (four out of 17).

## Discussion

To address a lack of guidance on how to write SP cases for communication training, we developed a curriculum for medical educators. This is the first educational intervention to examine the quality of written SP cases. This educational implementation suggests that our workbook is an effective method for teaching new medical educators how to write cases. Implementation of the newly developed workbook guiding case development significantly increased the quality of newly written SP cases compared to a previous lecture as assessed by a prespecified checklist of case criteria. While there was no significant difference in the ease of portrayal as assessed by the SP, this was likely due to sample size given (1) the large magnitude of difference between mean ratings and (2) the correlation found between the number of included case criteria and the SP evaluation.

This educational innovation has several strengths. It compared our curriculum to a previously existing curriculum via objective measures, including prespecified case criteria and blinded, independent SP evaluation. As guidance on this area is limited, the ability to compare to a previous curriculum bolsters support for the developed workbook. Furthermore, as SPs had been present in previous years to assist with case writing but were not present during this year's session, the demonstrated improvement in cases is more remarkable.

From a curricular standpoint, we utilized a flipped classroom model, requiring educators to complete the foundational work prior to the in-class session.^20^ This maximized the productivity of face-to-face time between the group and facilitator. Providing detailed guidance on case writing and an example case may additionally allow less experienced faculty to facilitate this session, even without SP input. Another strength is that applying this curriculum standardizes case writing and may, in turn, improve the quality and effectiveness of communication teaching.

This educational innovation should be considered with the following limitations. First, the total number of cases analyzed was small and from a single institution, limiting generalizability. Another consideration is that the cases were written in groups rather than by individuals. The total number of completed criteria with the new curriculum might have been fewer if educators had written their SP cases individually; however, we took the approach we did to mirror the previous teaching method in which cases were also written in groups, thereby maximizing the comparison between the new workbook and the previous lecture. Lastly, educator-reported confidence and attitudes were not available from the previous curriculum to compare responses.

Of note, while the checklist of case criteria has not undergone extensive validation, it was developed by communication educators with expert consensus, reflects components of the *MedEdPORTAL* guidelines for case submission, and demonstrated face validity when reviewed by educators and the SP. Assessing whether these criteria are necessary and sufficient for a quality SP case is an area for further research.

In conclusion, we developed a workbook that guides SP case development, paired with an in-person case-writing session to teach medical educators a skill vital to conducting effective communication skills education. The resulting curriculum significantly increased the quality of SP cases, with high reported educator confidence and satisfaction. This implementation supports further dissemination as well as research on the workbook's efficacy. In addition, it highlights the need for research to establish valid markers of high-quality SP cases.

## Appendices

SP Case Development Workbook.docxChecklist of 24 Case Criteria.docxPreclass Survey.docxPostclass Survey.docxFacilitator Guide.docx
All appendices are peer reviewed as integral parts of the Original Publication.
