# Supplementary material for: A Curriculum Innovation on Writing Simulated Patient Cases for Communication Skills Education
Source: MedEdPORTAL. 2021 Jan 12;17:11068. doi: 10.15766/mep_2374-8265.11068 (PMC7819616; doi:10.15766/mep_2374-8265.11068)
Supplement: Supplementary file 1 — SP Case Development Workbook.docxChecklist of 24 Case Criteria.docxPreclass Survey.docxPostclass Survey.docxFacilitator Guide.docx [file mep_2374-8265.11068-s001.zip › A. SP Case Development Workbook.docx]

Writing Simulated Patient Cases for Teaching Communication Skills

**Introduction**

Healthcare providers’ communication skills significantly impact the ability of patients and families to cope with illness.^1-5^ Multiple studies highlight the correlation between physician communication during new cancer diagnoses and patients’ subsequent adjustment.^1-3^ Effective communication enhances the physician-patient relationship and enables discussions where treatment plans align with patient goals.

In the last few decades, training with simulated patients has emerged as the leading method for instruction in communication skills.^6-12^ Through interactions with simulated patients (SPs), learners acquire skills ranging from taking a history of present illness to addressing goals of care. SPs, trained actors who portray patients in communication training, provide feedback through emotional or verbal reactions to learner statements. During teaching sessions with SPs, facilitators guide learners toward identifying common communication challenges and brainstorming ways these can be addressed. These teaching sessions allow for formative assessment to occur. In formative assessment, facilitators observe learners’ skill level and provide guidance for future learning.

While formative assessment is used to direct future performance, summative assessment evaluates learners’ current performance in order to assign a score or grade. *Standardized* patients, rather than simulated patients, are used for summative assessment^13-14^; they portray the patient in a uniform manner during structured clinical exams. Several guidelines currently exist for these cases.^15-19^

This curriculum will focus on how to write a *simulated* patient case for formative assessment and feedback. The case includes learning objectives, a summary of the patient the SP will portray, and instructions for learners and SPs. The curriculum has three parts: 1) case development from the educational goal to learner instructions, 2) case revision through personal assessment and collaboration with colleagues, and 3) requirements for publication.

Throughout the curriculum, instruction will be provided followed by an example case and then an opportunity to design your own case. In the example, Dr. Sergent, an internal medicine residency director, develops a communication case for her program. After each example of case development, we will ask you to write down how this task might apply to the learning opportunities in your own setting.

**Definitions**

1. **Teaching communication skills/communication training** – Formal didactic and experiential instruction on how to communicate with patients and/or families via speech or body language.
2. **Simulated patients (SPs)** – Trained actors who portray patients in communication training and give feedback to learners through emotional and/or verbal reactions to learner statements.
3. **Educational goal** – The overarching purpose of a case.
4. **Learning objectives** – Specific, measurable outcomes defining what knowledge, skills, or attitudes learners will acquire from a given case.
5. **Communication hurdles** – Learner communication challenges that are defined by the written case and enacted by SPs
6. **NURSE Statements** – Statements that express empathy by one of the following methods: *naming* the emotion, expressing *understanding*, *respecting* the person, *supporting* the person, and *exploring* the emotion.^20-21^ Refer to NURSE handout in resources.

**Overview**

**Steps of Writing a SP Case**

1. **Case Development**

Step 1 Educational Aims

Step 1A. Educational Goal

Step 1B. Learning Objectives

Step 1C. Communication Hurdles

Step 2 Clinical Situation

Step 2A. Basis for Patient Case

Step 2B. Clinical Diagnosis

Step 2C. Setting

Step 2D. Previous Experiences

Step 3 Patient History

Step 3A. Demographics

Step 3B. Medical History

Step 3C. Patient Characterization

Step 4 Case Instructions

Step 4A. General SP Instructions

Step 4B. Portrayal of Emotions

Step 4C. Learner Instructions

1. **Case Revision**

Step 1 Personal Review

Step 2 Review with Colleagues

Step 3 Case Criteria

1. **Publication**

**Checklist of Case Criteria**

**Resources**

Supplemental Material

Glossary

References

Personal Review

Review with Colleagues

Case Criteria

Step 1A

Educational Goals

Step 1B

Learning Objectives

Step 1C

Communication Hurdles

**Step 1**

**Educational Aims**

Step 2A

Basis for Case

Step 2B

Clinical Diagnosis

Step 2C

Setting

Consult Specialist

for

Medical Details

**Step 2**

**Clinical Situation**

Step 2D

Past Experiences

Step 3A

Demographics

**Step 3**

**Patient History**

Consult SP

for

Assess clarity of case

Step 4A

General Instructions

Step 4B

Portrayal of Emotions

**Step 4**

**Case Instructions**

Steps of Writing an SP Case

**Case Revision**

Step 3B

Medical History

Step 3C

Characterization

Step 4C

Learner Instructions

**Publication**

Case Development Tool

Efficacy Outcomes

Learner Satisfaction

**Case Development**

**Step 1A. Educational Goal**

Consider the following questions to guide case development.

1. *Why* is a new case needed?

Potential reasons include commonly-observed communication challenges, deficiencies in the current curriculum, accreditation mandates, and changing practices within medicine.

DR. SERGENT’S CASE

*Faculty on the general medicine wards report patients seem confused after residents deliver serious news and that residents often defer to the attending to deliver news. Dr. Sergent plans to develop a communication training session to teach internal medicine residents how to deliver serious news.*

WRITE: Why is a new case needed in your setting?

__________________________________________________________________________________

1. *Who* are the target learners?

Consider learners’ level of medical training, baseline communication skills, previous communication training, and the forum for case implementation (course, clerkship, rotation, national meeting).

DR. SERGENT’S CASE

*Dr. Sergent targets first-year internal medicine residents as they have the greatest amount of direct patient contact, frequently deliver serious news, and have a variety of communication skills training.*

WRITE: Who are your learners?

_Level of Learners:__________________________________________________________________ _Previous Communication Experience:__________________________________________________ ­ _Forum of Implementation:___________________________________________________________

1. *What* skills will the case address?

Examples include eliciting a HPI, responding empathically to emotion, and delivering serious news.

DR. SERGENT’S CASE

*Dr. Sergent focuses the case on delivering serious news using the Ask-Tell-Ask model.*

WRITE: What skills will your case address?

__________________________________________________________________________________

Combine the above answers to create the educational goal, a broad aim for the SP case.

DR. SERGENT’S CASE

*First-year residents will develop skills for communicating serious news via the Ask-Tell-Ask method.*

WRITE: What is the educational goal for your case? __________________________________________________________________________________

**Case Development**

**Step 1B. Learning Objectives**

Learning objectives delineate learner expectations at a granular level. We suggest two to four learning objectives per SP case.

Objectives must be SMART – 1) Specific, 2) Measurable, 3) Achievable, 4) Relevant, and 5) Time-Based. They are generally written in the following format:

Who will do how much of what by when?

1 1, 3 2 1, 3 5

where the numbers below indicate to which aspect of the SMART acronym they refer. A few examples of learning objectives are listed below; additional examples are provided in the Supplemental Material.

Table 1. Examples of Educational Goals and Associated Learning Objectives

| Educational Goals | Learning Objectives |
| --- | --- |
| Establishing rapport | Use non-verbal skills including sitting down and making eye contact |
| Obtaining a history | Illustrate at least three open-ended questions, employ one emptying question, use one summarizing statement in obtaining a history. |
| Delivering serious news | Use the Ask-Tell-Ask method, utilize a “warning shot,” use plain English. |
| Responding to emotion | Identify emotion and demonstrate an appropriate response with at least one of the following NURSE Statements^20-21^: naming the emotion, demonstrating respect for the person, and offering support. |

DR. SERGENT’S CASE

By the end of the session, first year medicine residents will be able to:

1. Assess patient understanding prior to delivering serious news.
2. Use clear and simple terms (avoid jargon) in delivering serious news.
3. Respond to patient emotion by using a minimum of two NURSE statements.

WRITE: List two to three learning objectives for your case.

____________________________________________________________________________________________________________________________________________________________________________________________________________________________________________________________________________________________________________________________________________________________________________________________________________________________________________________________________________________

**Case Development**

**Step 1C. Communication Hurdles**

Communication hurdles are communication challenges based upon the learning objectives. They are defined by the written case and enacted by SPs, proving the learner opportunities to practice specific skills. Inclusion of several communication hurdles allows for a variety of learner skill within the same academic level. Most learners may accomplish the first hurdle, a half may finish the second, and a handful of skilled learners may complete all of them.

DR. SERGENT’S CASE

*After the learner has given the diagnosis in clear language, the SP will react with emotion. If the learner is skilled and responds to the emotion by using NURSE statements (such as naming the emotion and indicating understanding), the SP will deescalate his emotion. If the learner is unskilled and provides premature reassurance, continues to reaffirm the diagnosis, or attempts to move on to next steps without addressing the emotion, the SP will make emotions more obvious.*

WRITE: Describe an appropriate communication hurdle for your case.

______________________________________________________________________________________________________________________________________________________________________________________________________________________________________________________________________________________________________________________________________________________________________________________________________________________________________________________________________________________________________________________________________________________________________________________________________________________________________________________________________________________________________________________________

**Case Development**

**Step 2A. Basis for Patient Case**

The clinical situation of the case should stem from the previously defined goals and objectives. We recommend writing the case with at least one previous patient in mind. Past cases are beneficial as they: 1) contribute a sense of realism, 2) decrease effort spent on brainstorming a past history, and 3) enhance internal consistency. Certainly, patient-identifying details must be excluded from the case.

**Step 2B. Clinical Diagnosis**

While curricular requirements or clinical needs may determine the patient’s clinical diagnosis, other times you have flexibility. When delivering serious news, does the patient have cancer or diabetes? If a cancer diagnosis, does the patient have Stage II breast cancer or Stage IV melanoma with brain metastases? These decisions affect learner perception, patient reaction, and challenge level of the case.

When selecting a diagnosis, consider its relevance to the learner. The case should be a routine, easily-recognizable situation; that way, they can focus on the communication task rather than medical details. Additionally, the more specialized the learner, the more a case needs to align with his or her area of expertise. For topics outside your area, it is critical to solicit input from a content expert; the expert may identify the clinical situation most likely to meet your objectives and provide details on standard of care.

**Step 2C. Setting**

The setting should match where the situation most arises or where educational need is greatest. Potential settings include the hospital, intensive care unit, clinic, nursing facility, and a patient’s home.

**Step 2D. Previous Experiences**

A SP case can be written as either an initial visit with a patient or a follow-up visit. Even for first visits, however, the patient has had interactions with others in the healthcare system. These previous interactions may shape the medical knowledge and emotion the patient brings to the case.

DR. SERGENT’S CASE

*Dr. Sergent creates a case on delivering a new diagnosis of metastatic lung cancer based on a similar case she encountered. She sets the case in the hospital where she and her colleagues note the residents’ communication challenges. The clinical diagnosis of the patient is one of the most common, serious diagnoses given on the general medicine wards. The intern who will deliver the diagnosis admitted the patient last night and discussed obtaining a CT for further evaluation. Prior to this admission, the patient had not visited a physician in over 50 years.*

WRITE: Provide details for your case below.

_Basis for Patient Case:___________________________________________________________ _Clinical Diagnosis:_______________________________________________________________ _Setting:_______________________________________________________________________ _Relationship to the Patient/Past Experiences:_________________________________________ ______________________________________________________________________________

**Case Development**

**Step 3. Patient History**

The patient’s history includes demographics, medical history, and patient characterization. When writing the patient history, consider:

1. *Realism* – Target common or easily-recognizable situations; treatment history must be based on the current standard of care.
2. *Relevance* – The details of a case must align with the learner’s area of practice.
3. *Learner Skill Level* – The skill level encompasses not only the learner’s level of training, but also their previous experience with communication skills training and their own practice habits.
4. *Internal Consistency* – The personality of the patient and the history needs to be consistent.

**Step 3A. Demographics**

Demographics include the following: age range, gender, ethnicity, and chief concern/diagnosis. You may need to adjust demographics based on either the educational goals or the group of available SPs. To allow for a broader range of SPs to play the case, you may not need to specify gender or ethnicity.

**Step 3B. Medical History**

While every element of the history should be addressed in each case (HPI, ROS, PMH, SHx, Fam Hx, meds), some elements will be more important than others based on the learning objectives and communication hurdles. When writing the history, it is helpful to brainstorm questions the SPs should be able to answer and ensure this information is provided. Ask SPs for their input early.

The history includes not only the presenting symptoms, but more importantly, the broader story of the patient. How did he or she come to this clinical encounter and with whom has he or she interacted along the way? What emotions does he or she bring to the encounter? For instance, a patient receiving treatment for cancer may believe that the diagnosis was delayed and subsequently distrust physicians. The SP can subsequently deepen and adjust the interpretation of this incident, including addition of medical details.

**Step 3C. Patient Characterization**

Patient characterization includes the patient’s overall appearance (i.e., disheveled, neat, wearing a bandana, in a hospital gown), demeanor (i.e., initially angled away from interviewer, asking several questions without awaiting a response), and details about medical literacy. You may consider props such as a wheelchair or glasses or make-up to portray a bruise or rash.

*Complexity*

While specific details add realism, too much detail can overwhelm the learner and distract from the learning objectives in the encounter. Work toward a balance, and consider the ability to present variable levels of detail to the SP versus the learner, to be discussed in Step 4.

DR. SERGENT’S CASE

*DEMOGRAPHICS: Mr. Greg Hanson is a 64-year-old Caucasian man with 3 months of progressively worsening productive cough.*

*HPI: He has also had mild, progressive shortness of breath while climbing stairs, which he attributes to smoking and increasing age. Over the past month, his sputum has become blood-tinged. He presented to the emergency department yesterday afternoon after coughing up a tablespoon of blood.*

*ROS: While he has not weighed himself, he has had to cinch his belt 1-2 holes tighter in the last few weeks. He has also had mild fatigue when brushing his horses. He has not had any fevers, night sweats, abdominal pain, swelling, or other symptoms.*

*PMH: He has smoked 1.5 packs per day over 50 years (since age 14). He has not seen a doctor since receiving a tetanus shot at twelve years old and has no other diagnoses. No previous hospitalizations.*

*Social History: He lives alone on his family farm about 45 minutes away. He used to farm wheat and corn, but retired three years ago. Still keep a couple horses. He has an older brother who lives close by, but whom he has not seen in twenty years due to an argument over farming together. A younger sister lives in Oregon whom he last spoke with this past Christmas. No close friends, prefers solitude. He doesn’t consider himself religious.*

*Family History: Unknown if either his brother or sister have medical issues. Father died from a heart attack in his 50s, mother died from “old age” in her 80s.*

*Medications: None, no over the counter supplements or herbs*

*Patient Characterization: Wears a hospital gown, slightly gruff with tussled hair, makes eye contact. He has poor medical literacy.*

*Medical Management to Date: In the emergency room, a chest x-ray showed that “something was wrong” including “fluid on the lung.” He was admitted to the hospital for further evaluation and chest CT overnight. The following morning, he is awaiting the results of the scan, which showed probable lung cancer with spread to the liver.*

WRITE: Detail your patient’s history.

_DEMOGRAPHICS:______________________________________________________________________________________________________________________________________________HPI:________________________________________________________________________________________________________________________________________________________________________________________________________________________________________________________________________________________________________________________________________________________________________________________________________________________________________________________________________________________________________________________________________________________________________________________________________________________________________________________________________________________________________________________________________________________________________________________________________ __ROS:_____________________________________________________________________________________________________________________________________________________________________________________________________________________________________Past Medical History (past treatments, past hospitalizations, previous interactions with______ _learner/medical team):__________________________________________________________ _____________________________________________________________________________________________________________________________________________________________________________________________________________________________________________________________________________________________________________________________________________________________________________________________________________________________________________________________________________________Social History (current living situation, work history, family/friends involved, relevant patient__ _values, applicable drug use history):________________________________________________ _________________________________________________________________________________________________________________________________________________________________________________________________________________________________________________________________________________________________________________________Family History (past family medical history that may impact perceptions/values):____________ ___________________________________________________________________________________________________________________________________________________________________________________________________________________________________________Medications:__________________________________________________________________________________________________________________________________________________Patient Characterization: ­­­_________________________________________________________ ______________________________________________________________________________ _______________________________________________________________________________Medical Management to Date:____________________________________________________ ______________________________________________________________________________ ____________________________________________________________________________________________________________________________________________________________ ______________________________________________________________________________

**Case Development**

**Step 4A. General SP Instructions**

The SP instructions discussed in this 4A and 4B are provided to the SPs in addition to the patient history written in Step 3. There are both general SP instructions that apply to all cases and specific guidelines for emotional portrayal of a patient.

If you are working with professional SPs, they will likely be familiar with the general standards; however, for the first time collaborating, you may want to review the following guidelines, adapted from *The Comprehensive Textbook of Healthcare Simulation*.

Table 2. General SP Guidelines

| Do: |
| --- |
| - Maintain the role throughout the encounter |
| - Try to feel, think, and react like the patient |
| - Seek clarification as needed from the author |
| Don’t: |
| - Break from the role |
| - View the case as a script to be memorized |

**Case Development**

**Step 4B. Portrayal of Emotions**

Emotional responses to learner statements will differ based on whether the student is using skilled or unskilled communication techniques. For unskilled learner responses, SPs make the emotion more obvious, while for skilled responses, SPs deescalate emotion and provide more information. Examples of unskilled techniques are provided in the Supplemental Material.

It is critical that SPs have freedom in interpreting the patient’s reaction to the learners. At the same time, guidance of emotional portrayal may be provided based on the learning objectives and the learners’ baseline skill level. For learners with no prior training or limited communication skills, highly emotional situations can be overwhelming; you may need to specify limits on emotional intensity.

When SPs are portraying emotion, there are five aspects to consider:

- Type of Emotions – A patient or family member may have several emotions; however, for learners who are less skilled at identifying emotion, it is better to express only one emotion at a time.
- Timing of Emotions – Emotions may be triggered by new information or by the learner’s words and actions during the interaction.
- Method of Displaying Emotions – This includes language, tone of voice, and body language. This is typically at the discretion of the SP, but you can suggest questions or statements SPs may make during the encounter.
- Emotional Intensity – Emotional intensity should depend on the skill level of the learner. For instance, is crying or yelling appropriate?
- Response to Learner – The response includes when and to what extent the SP deescalates an emotion.

DR. SERGENT’S CASE

*Learning Objective: First-year medicine residents will utilize a minimum of two NURSE statements in responding to patient emotion.*

*Portrayal of Emotion:*

*Upon the diagnosis, the SP will convey disbelief. Examples of this may include crossing arms, turning away, or expressing distrust of the test.*

*Unskilled Response:*

*If the learner uses unskilled techniques such as providing premature reassurance, continuing to reaffirm the diagnosis, or attempting to move on to next steps without addressing the emotion, the SP will make the emotion more obvious. This may include speaking in a louder voice (no yelling) or shifting in the chair.*

*Skilled Response*

*If the learner uses skilled techniques to respond to the emotion such as use of NURSE statements, the SP will deescalate his emotion. Examples of deescalating emotion include decreasing volume of speech and providing more information.*

WRITE: Expand upon a time in your case when the patient may have an emotional response.

_Portrayal of Emotion:____________________________________________________________ _____________________________________________________________________________________________________________________________________________________________Unskilled Response:_____________________________________________________________ ____________________________________________________________________________________________________________________________________________________________ _______________________________________________________________________________Skilled Response:_______________________________________________________________ __________________________________________________________________________________________________________________________________________________________________________________________________________________________________________

**Case Development**

**Step 4C. Learner Instructions**

After completing the SP instructions, you will need to write an introduction to the case for the learners. The introduction may include the diagnosis, a brief summary of clinical situation, whether the learner has previously met the patient, and the goal for the encounter. While the amount of information on the purpose of the encounter may vary, avoid explicitly stating the learning objectives of the case.

DR. SERGENT’S CASE

*Case: Mr. Greg Hanson is a 64-year-old man with a 75 pack-year smoking history who presented to the emergency room yesterday afternoon with a tablespoon of hemoptysis. He had a three-month history of worsened productive cough, weight loss, and shortness of breath. A chest x-ray showed a right lower lobe opacity, a moderate pleural effusion, and mediastinal lymphadenopathy. He was admitted to your general medicine team for evaluation, and you ordered a chest CT. The CT scan overnight showed a lung mass concerning for malignancy and nodules in the liver consistent with metastatic disease.*

*Task: Deliver news of the CT scan to Mr. Hanson.*

WRITE: What instructions will you provide to your learners?

_Case:_________________________________________________________________________ _____________________________________________________________________________________________________________________________________________________________________________________________________________________________________________________________________________________________________________________________________________________________________________________________________________________________________________________________________________________________________________________________________________________________________________________________________________________________________________________________________________________________________________________________________________________________________________________________________________Task:_______________________________________________________________________________________________________________________________________________________

**Case Revision**

Once you complete the first draft of the case, it is important to review and revise the case. This includes both a personal assessment of the case and a review with colleagues.

**Step 1. Personal Review**

Consider:

- Is the context of the encounter realistic?
- Is the encounter consistent with current medical practices?
- Is the encounter appropriate for the leaners’ skill level?
- Does the encounter focus on communication skills that are necessary and important?
- How does the case fit with the rest of the curriculum? Does it emphasize previously-encountered concepts or serve as an initial introduction to these concepts?

**Step 2. Review with Colleagues**

The following is a list of potential reviewers for the case along with reasons to consider including them in a review.

Table 3. List of Reviewers

| Reviewers | Reasons for Review |
| --- | --- |
| Medical specialist (i.e., oncologist, thoracic surgeon) | Clarify medical details |
| Communication educator | Evaluate case’s ability to meet learning objectives |
| Recent learners | Assess appropriateness for learner level |
| Patients or families | Evaluate for realism |
| Simulated patients | Assess clarity of case, realism of patient reactions |

**Step 3. Case Criteria**

During revision, confirm you have included all the criteria for a SP case. See the next page for a checklist detailing necessary elements.

**Publication**

Publication requires an increased level of detail for reproducibility purposes. MedEdPORTAL, run by the Association of American Medical Colleges, is an online repository of peer-reviewed curricula and educational material. It is an ideal venue for publication of SP cases. When preparing SP cases for submission, MedEdPORTAL requires the use of the Portal Standardized Patient Case Development Tool. For further details on submission, see <https://www.mededportal.org/authors/>.

To publish in MedEdPORTAL, you will need to assess the efficacy of a new case. This may include comparing it to a prior case or to a previous curriculum that lacked a SP case. Your study should include not only learner satisfaction but also higher-level outcomes measuring its effectiveness.^22^

**Checklist of Case Criteria**

*Educational Aims*

- Educational goal
- 2-4 learning objectives
- 2-4 communication hurdles
- Level of learner

*Case Information*

Introductory Information

- - Name
  - Demographics
  - Chief Complaint/Diagnosis

HPI

- - Onset of symptoms
  - Progression of symptoms
  - Understanding of the situation

Past Medical History

- - Previous treatments
  - Past hospitalizations
  - Previous interaction(s) with learner/treatment team

Social History

- - Work history
  - Current living situation
  - Friends/family involved
  - Relevant values

Family History

- - Similar family situations in the past

Patient Characterization

- - Appearance
  - Behavior
  - Medical Literacy

*Case Instructions*

Portrayal of Emotion

- - Unskilled response
  - Skilled response
- Learner Instructions

**Resources**

**Supplemental Material**

Table 4. NURSE Statements for Articulating Empathy*

| Statements | Example |
| --- | --- |
| Naming | “You seem disappointed.”  “This is a frustrating situation.” |
| Understanding | “This helps me understand what you’re thinking.”  “I can’t imagine what this must be like for you.” |
| Respecting | “I can see you have really been trying to follow our instructions.”  “I can see how hard you’re working to advocate for your mom.” |
| Supporting | “I will do my best to make sure you have what you need.”  “I will check in with you tomorrow.” |
| Exploring | “Can you tell me more about what you mean by…” |

*Adapted from http://vitaltalk.org/guides/responding-to-emotion-respecting/

Table 5. Educational Goals and Associated Learning Objectives

| Educational Goals | Learning Objectives |
| --- | --- |
| Establishing rapport | Use non-verbal skills including sitting down and making eye contact |
| Obtaining a history | Illustrate at least three open-ended questions, employ one emptying question, use one summarizing statement in obtaining a history |
| Delivering serious news | Use the Ask-Tell-Ask method, utilize a “warning shot,” speak in clear and simple terms (plain English) |
| Disclosing a medical error | Offer an apology, make a direct explanation of the error, avoid shifting blame, explain plan to prevent similar errors in the future |
| Responding to emotion | Identify emotion and demonstrate an appropriate response with at least one of the following: naming the emotion, demonstrating respect for the person, and offering support |
| Reframing the clinical situation | Use the Ask-Tell-Ask method, state one to two sentence headline without jargon, pause for response, check for understanding |
| Addressing questions of prognosis | Confirm whether questions on prognosis are cognitive or emotional by verifying the patient is ready to discuss prognosis |
| Making a recommendation | Demonstrate respect by asking permission to make the recommendation, summarize the previous discussion, and describe how the recommendation aligns with the patient’s/family’s goals |

Table 6. Examples of Unskilled Communication Techniques

| Unskilled Techniques | Example |
| --- | --- |
| Jargon | “The CT scan showed a pulmonary embolus.” |
| Interrupting | Patient: “I’ve been feeling down over…”  Physician: “Have you been thinking of hurting yourself?” |
| Lecturing | “We got the test results back. The scan showed the cancer has spread to the liver. So, we are going to switch treatments. We will start…” |
| Premature Reassurance | Family member: “What if the antibiotics don’t work?”  Physician: “They will work. Your mom will be fine.” |

**Resources**

**Glossary**

Blocking

*An unskilled communication technique whereby the physician does not respond to a patient’s concern. This may be used to avoid further discussion of the concern.*

Communication hurdles

*Learner communication challenges that are defined by the written case, enacted by SPs, and to which learners must respond appropriately to advance in the case.*

Educational goal

*The overarching purpose of a curriculum.*

Emptying Question

*A concluding question which invites the patient to mention any additional information. Typically of the format: “What other (concerns, questions, thoughts) do you have regarding…”*

Formative Assessment

*A procedure whereby a teacher elicits evidence of student achievement for purposes of providing guidance on further learning.*

Jargon

*Terminology that is difficult for the lay person to understand.*

Learning objectives

*Specific, measurable outcomes defining what knowledge, skills, or attitudes learners will acquire from a given curriculum.*

Lecturing

*An unskilled communication technique where the physician delivers a large amount of information without allowing a patient to respond.*

NURSE Statements

*Statements that express empathy by one of the following methods: naming the emotion, expressing understanding, respecting the person, supporting the person, and exploring the emotion. Refer to NURSE Statements in Supplemental Materials for examples.*

Plain English

*Clear, easy to understand language that avoids jargon; also known as “layman’s terms.” Use of plain English is a skilled communication technique.*

Premature Reassurance

*An unskilled communication technique in which the physician promptly responds to a patient’s concerns with encouragement and without exploring the concerns.*

Simulated patients (SPs)

*Trained actors who portray patients in communication training and provide feedback to learners through emotional and/or verbal reactions to learner statements.*

Standardized Patients

*Trained actors who portray patients in a uniform manner for assessment purposes.*

Summative Assessment

*Evaluation of a learner for purposes of determining a score or grade.*

Teaching communication skills/communication training

*Formal didactic and experiential instruction on how to communicate with patients and/or families via speech or body language.*

**Resources**

**References**

1. Last BF, van Veldhuizen AM. Information about diagnosis and prognosis related to anxiety and depression in children with cancer aged 8-16 years. *Eur J Cancer*. 1996.
2. Roberts CS, Cox CE, Reintgen DS, Baile WF, Gibertini M. Influence of physician communication on newly diagnosed breast cancer patients’ psychologic adjustment and decision-making. *Cancer*. 1994;74:336-41.
3. Slavin LA, O’Malley JE, Koocher GP, Foster DJ. Communication of the cancer diagnosis to pediatric patients: impact on long-term adjustment. *Am J Psychiatry*. 1982;139:179-83.
4. Baile W, Buckman R, Lenzi R, Glober G, Beale E, Kudelka A. SPIKES–A six-step protocol for delivering bad news: application to the patient with cancer. *Oncologist*.2000.
5. Wright A, Zhang B, et al. Association between end-of-life discussions, patient mental health, medical care near death, and caregiver bereavement adjustment. *JAMA*. 2008.
6. Gysels MH, Richardson A, Higginson IJ. Communication training for health professions who care for patients with cancer: a systematic review of training methods. *Support Care Cancer*. 2005;13(6):356-66.
7. Back AL, Arnold RM, Baile WF, et al. Efficacy of communication skills training for giving bad news and discussing transitions to palliative care. *Arch Intern Med*. 2007;167.
8. Bays AM, Engelberg RA, Back AL, et al. Interprofessional communication skills training for serious illness: evaluation of a small-group, simulated patient intervention. *J Palliat Med*. 2014;17(2):159-66.
9. Clayton JM, Butow PN, Waters A, et al. Evaluation of a novel individualized communication-skills training intervention to improve doctors’ confidence and skills in end-of-life communication. *Palliat Med*. 2012;27(3):236-43.
10. Howley LD. Performance assessment in medical education: where we’ve been and where we’re going. *Eval Health Prof*. 2004;27(3):285-303.
11. Levine AL, DeMaria Jr. S, Schwartz AD, Sim AJ (Eds). *The Comprehensive Textbook of Healthcare Simulation*. Springer-Verlag. New York: 2013.
12. Berkhof M, van Rijssen HJ, Scholar AJM, Anema JR, van der Beek AJ. Effective training strategies for teaching communication skills to physicians: An overview of systematic reviews. Patient Educ Couns. 2011 Aug;84(2):152-62.
13. Stillman PL et. al. Assessing clinical skills of residents with standardized patients. Ann Intern Med. 1986;105(5):762-71.
14. Barrows HS. An overview of the uses of standardized patients for teaching and evaluating clinical skills. Acad Med. 1993;68(8): 443-53.
15. King AM, Perkowski-Rogers LC, Pohl HS. Planning standardized patient programs: case development, patient training, and costs. Teaching and Learning in Medicine. 1994;6(1):6-14.
16. Advanced Clinical Education Center. Standardized patient case template. University of Pittsburgh School of Medicine.
17. Olive KE, Elnicki DM, Kelley MJ. A practical approach to developing cases for standardized patients. Advances in Health Sciences Education. 1997;2:49-60.
18. Wilson Centre for Research in Education. A guide for writing a standardized patient case. University of Toronto.
19. Scott CS, Brannaman V, Struijk J, Ambrozy D. Standardized patient case development workbook. University of Washington School of Medicine; 1999.
20. Back AL, Arnold RM, Baile WF, Fryer-Edwards KA. Approaching difficult communication tasks in oncology. CA Cancer J Clin. 2005 May-June;55(3):164-77.
21. Smith RC. Patient-Centered Interviewing: An Evidence-Based Method. Philadelphia, PA. Lippincott, Williams, & Wilkins 2002.
22. Kirkpatrick DL, Kirkpatrick JD. Evaluating training programs: the four levels. 3rd ed. San Francisco: Berrett-Koehler Publishing Company; 2006.
