# Supplementary material for: A Curriculum Innovation on Writing Simulated Patient Cases for Communication Skills Education
Source: MedEdPORTAL. 2021 Jan 12;17:11068. doi: 10.15766/mep_2374-8265.11068 (PMC7819616; doi:10.15766/mep_2374-8265.11068)
Supplement: Supplementary file 1 — SP Case Development Workbook.docxChecklist of 24 Case Criteria.docxPreclass Survey.docxPostclass Survey.docxFacilitator Guide.docx [file mep_2374-8265.11068-s001.zip › B. Checklist of 24 Case Criteria.docx]

Checklist of 24 Case Criteria for Written SP Cases

| **Educational Aims** |
| --- |
|  |
| - Educational goal |
| - 2-4 learning objectives - At least one communication hurdle |
| - Training level of learner |
|  |
| **Case Information** |
|  |
| *Introductory Information* |
| - SP name |
| - Demographics |
| - Chief complaint or diagnosis |
|  |
| *HPI* |
| - Onset of symptoms |
| - Progression of symptoms |
| - Patient understanding of the situation |
|  |
| *Past Medical History* |
| - Previous treatments |
| - Past hospitalizations |
| - Previous interaction(s) with learner or other healthcare providers |
| *Social History* |
| - Work history |
| - Current living situation |
| - Family/friend involvement |
| - Patient values relevant to decision-making |
|  |
| *Family History* |
| - Family history |
|  |
| *Patient Characterization* |
| - Patient appearance |
| - Patient behavior |
| - Patient’s medical literacy |
|  |
| **Case Instructions** |
|  |
| *Portrayal of Emotion* |
| - Portrayal of emotion with unskilled response |
| - Portrayal of emotion with skilled response |
|  |
| - Learner instructions |
