# Supplementary material for: A Curriculum Innovation on Writing Simulated Patient Cases for Communication Skills Education
Source: MedEdPORTAL. 2021 Jan 12;17:11068. doi: 10.15766/mep_2374-8265.11068 (PMC7819616; doi:10.15766/mep_2374-8265.11068)
Supplement: Supplementary file 1 — SP Case Development Workbook.docxChecklist of 24 Case Criteria.docxPreclass Survey.docxPostclass Survey.docxFacilitator Guide.docx [file mep_2374-8265.11068-s001.zip › C. Preclass Survey.docx]

Pre-Class Survey on Simulated Patient Case Curriculum

This survey will ask about your previous experiences with writing simulated patient cases. Please sign this cover sheet to indicate your consent to participate. When we receive your survey, we will permanently separate this cover sheet from your survey so that your responses will be anonymous and confidential.

Thank you for your input!

SIGNATURE ____________________________________________________________

PRINTED NAME ____________________________________________________________

*I understand that my signature indicates my consent to participate in this survey. This signature page will be detached from my responses so that they will remain anonymous.*

Pre-Class Survey on Simulated Patient Case Curriculum

For the survey, SP is used for simulated patients, trained actors who portray patients in communication training and give feedback to learners through emotional and/or verbal reactions to learner statements.

To connect this survey with the post-class survey, **please write** the first letter of the city where you were born and the last four digits of your phone number below:

First letter of the city where you were born: ____

Last four digits of your phone number: ____ ____ ____ ____

**PREVIOUS INSTRUCTION ON WRITING A SP CASE**

1. **Before today**, have you ever received instruction on how to write a SP case?
   - Yes
   - No

*If no, continue to Question 3 in Section 2.*

**Please answer the following question on a 1-5 scale.**

| 1  Strongly Disagree | 2 | 3  Neither Agree nor Disagree | 4 | 5  Strongly Agree |
| --- | --- | --- | --- | --- |

1. I am satisfied with the instruction I have received on how to write a SP case.

__________

**PREVIOUS COMMUNICATION TEACHING WITH A SP**

1. **Before this class**, have you taught communication skills utilizing a SP?

- Yes
- No

*If No, continue to Question 5 in Section 3.*

1. **Before this class**, to which groups of learners have you taught communication skills utilizing a SP? Check all that apply.
   - Medical students
   - Residents
   - Fellows
   - Physician Attendings
   - Other, please list: ______________________________

**PREVIOUS EXPERIENCE WRITING A SP CASE**

1. **Before today**, have you ever written a SP case from scratch to either teach or evaluate learners?
   - Yes
   - No
2. How many SP cases have you **written from scratch** before today?
   - N/A
   - 1-2
   - 3-4
   - 5 or more
3. **Before today**, have you ever modified an existing SP case to either teach or evaluate learners?
   - Yes
   - No
4. How many SP cases have you **modified** before today?
   - N/A
   - 1-2
   - 3-4
   - 5 or more
5. Who were your target learners for the SP case(s) you have written or modified? Check all that apply.
   - N/A
   - Medical students
   - Residents
   - Fellows
   - Physician Attendings
   - Other, please list: __________________________________

**ATTITUDES – IMPORTANCE AND CONFIDENCE**

**Please answer the following three questions on a 1-5 scale.**

| 1  Strongly Disagree | 2 | 3  Neither Agree nor Disagree | 4 | 5  Strongly Agree |
| --- | --- | --- | --- | --- |

1. The ability to write SP Cases is an important skill for clinician educators to have.

__________

1. Writing SP cases will be part of my future work as a clinician educator.

__________

1. I am confident in my ability to write SP cases for teaching or evaluating communication skills.

__________
