# Supplementary material for: A Curriculum Innovation on Writing Simulated Patient Cases for Communication Skills Education
Source: MedEdPORTAL. 2021 Jan 12;17:11068. doi: 10.15766/mep_2374-8265.11068 (PMC7819616; doi:10.15766/mep_2374-8265.11068)
Supplement: Supplementary file 1 — SP Case Development Workbook.docxChecklist of 24 Case Criteria.docxPreclass Survey.docxPostclass Survey.docxFacilitator Guide.docx [file mep_2374-8265.11068-s001.zip › D. Postclass Survey.docx]

Post-Class Survey on Simulated Patient Case Curriculum

This survey will ask about your experiences with the workbook on writing simulated patient cases and the in-class session from today. We are asking you these questions to identify ways to improve the curriculum for future learners.

Please sign this cover sheet to indicate your consent to participate. When we receive your survey, we will permanently separate this cover sheet from your survey so that your responses will be anonymous and confidential.

Thank you for your input!

SIGNATURE ____________________________________________________________

PRINTED NAME ____________________________________________________________

*I understand that my signature indicates my consent to participate in this survey. This signature page will be detached from my responses so that they will remain anonymous.*

Post-Class Survey on Simulated Patient Case Curriculum

For the survey, SP is used for simulated patients, trained actors who portray patients in communication training and give feedback to learners through emotional and/or verbal reactions to learner statements.

To connect this survey with the pre-class survey, **please write** the first letter of the city where you were born and the last four digits of your phone number below:

First letter of the city where you were born: ____

Last four digits of your phone number: ____ ____ ____ ____

**FEEDBACK ON THE CURRICULUM**

1. When writing the SP case in class, which section of the case-writing was **most** challenging? Please select one answer.
   - None were challenging
   - Step 1 – Educational Aims
   - Step 2 – Clinical Situation
   - Step 3 – Patient History
   - Step 4 – Case Instructions
   - All were equally challenging
2. Why was the section selected in Question 1 challenging? Please write N/A if none were challenging.

________________________________________________________________________________________________________________________________________________________________________________________________________________________________________________________________________________________________________________________

1. How would you improve the workbook? Please list suggestions below.

________________________________________________________________________________________________________________________________________________________________________________________________________________________________________________________________________________________________________________________

1. How would you improve the in-class session? Please list suggestions below.

________________________________________________________________________________________________________________________________________________________________________________________________________________________________________________________________________________________________________________________

**ATTITUDES – IMPORTANCE AND CONFIDENCE**

**Please answer the following questions on a 1-5 scale.**

| 1  Strongly Disagree | 2 | 3  Neither Agree nor Disagree | 4 | 5  Strongly Agree |
| --- | --- | --- | --- | --- |

1. The ability to write SP cases is an important skill for clinician educators to have.

__________

1. Writing SP cases will be part of my future work as a clinician educator.

__________

1. I am confident in my ability to write SP cases for teaching or evaluating communication skills.

__________

­­­

**ATTITUDES – CURRICULUM**

**Please answer the following questions on a 1-5 scale.**

| 1  Strongly Disagree | 2 | 3  Neither Agree nor Disagree | 4 | 5  Strongly Agree |
| --- | --- | --- | --- | --- |

1. The curriculum (including the workbook and the in-class session) effectively prepared me to write a SP case.

__________

1. The example SP case used throughout the workbook effectively illustrated the key concepts of SP case writing.

__________

1. The workbook was clearly written.

__________

1. The workbook steps were easy to follow.

__________

1. The in-class session was an essential component to my learning how to write a SP case.

__________

1. I am more likely to write SP cases in the future as a result of this curriculum.

__________
