# Supplementary material for: A Curriculum Innovation on Writing Simulated Patient Cases for Communication Skills Education
Source: MedEdPORTAL. 2021 Jan 12;17:11068. doi: 10.15766/mep_2374-8265.11068 (PMC7819616; doi:10.15766/mep_2374-8265.11068)
Supplement: Supplementary file 1 — SP Case Development Workbook.docxChecklist of 24 Case Criteria.docxPreclass Survey.docxPostclass Survey.docxFacilitator Guide.docx [file mep_2374-8265.11068-s001.zip › E. Facilitator Guide.docx]

Facilitator Guide

The educational goal of this curriculum is for medical educators to gain experience writing effective SP cases for communication skills training.

Educational Objectives

By the end of the module, participating medical educators will be able to:

1. Describe the four steps to writing a simulated patient case.
2. Write 2-3 learning objectives for a new case that meet all SMART criteria.
3. In coordination with 3-4 other medical educators, develop a new simulated patient case that fulfills at least 22 of the 24 criteria.
4. Describe the process of case review prior to implementation in an educational setting.

Previous Class Session Agenda

Provide educators with the workbook 3-7 days prior to the case-writing session to ensure sufficient time for review and to craft ideas for a potential case.

10 min Introduce the purpose of the SP case-writing session

Provide with electronic/hard copies of the case-development workbook

Ask students to review the workbook and draft case ideas for the next session

In-Class Session Agenda

10 min Check in and answer questions on the workbook or SP case-writing

5 min Educators divide into groups of 4-5 based on intended learner level (med student, resident, fellow, etc) and case topic

55 min Educators work on Step 1 through Step 3B

15 min Break

40 min Educators work on Step 3C through Step 4

5 min Regroup

35 min Debrief

Debrief

During the debrief, have the educators reflect on the process of case writing. For instance, what challenges did they experience? One goal is for the class to identify common pitfalls in the writing process. Similarly, have groups share insights they learned that may address these challenges. Potential questions for the debrief include:

1. How did the case writing go?
2. What was the easiest part of writing the case?
3. What was the hardest part of writing the case? How did you work through this challenge?
4. What was most surprising to you about this process?
5. How did your target learner influence your writing?
6. What did you do to make the case realistic?
7. Are there resources or experts that you wished were available during the process?
8. How did writing the communication hurdles go?
9. What did you learn from this exercise?

Tips for Debrief

Explore the how incorporating details from real patient experiences affects the written case. Does it make the case realistic? On the other hand, does providing too many details become burdensome to the SP or distract from the goal of the communication encounter?

If class time allows, consider involving a SP to portray a patient from a newly written case. An educator who did not write the case should take on the learner role. After the interaction, have the group who wrote the case reflect with the rest of the class on how the interaction unfolded – did it go as expected? Did the learner become sidetracked on extraneous information? Looking back, was the information provided to the learner at the beginning of the encounter meaningful and sufficient?
